# Supplementary material for: Association between Early Immune-Related Adverse Events and Survival in Patients Treated with PD-1/PD-L1 Inhibitors
Source: J Clin Med. 2023 Jan 17;12(3):736. doi: 10.3390/jcm12030736 (PMC9917734; doi:10.3390/jcm12030736)
Supplement: Supplementary file 1 [file jcm-12-00736-s001.zip › jcm-2109268-supplementary.pdf]

Supplementary Table S1. Search terms

The following terms were searched in [Title/Abstract]: (“nivolumab”[Supplementary Concept] OR “Nivolumab”[tiab] OR “Opdivo”[tiab] OR “MDX-1106”[tiab] OR “ONO-4538”[tiab] OR “BMS-936558”[tiab] OR “NIVO”[tiab] OR “pembrolizumab”[Supplementary Concept] OR “pembrolizumab”[tiab] OR “lambrolizumab”[tiab] OR “keytruda”[tiab] OR “MK-3475”[tiab] OR “SCH 900475”[tiab] OR “avelumab”[Supplementary Concept] OR “Avelumab”[tiab] OR “MSB0010718C”[tiab] OR“MPDL3280A”[Supplementary Concept] OR “MPDL3280A”[tiab] OR “atezolizumab”[tiab] OR “Tecentriq”[tiab] OR “RG7446”[tiab] OR “RO5541267”[tiab] OR “Durvalumab”[tiab] OR “MEDI4736”[tiab] OR “MEDI-4736”[tiab]OR checkpoint inhibitor*[tiab] OR “PD-1”[tiab] OR “PDL1”[tiab]OR “immunotherapy”[tiab] OR “immune checkpoint”[tiab]) AND(“Drug-Related Side Effects and Adverse Reactions” [MeSH Terms] OR “Side Effects of Drugs”[tiab] OR “Drug Side Effects”[tiab] OR “Drug Side Effect”[tiab] OR “Effects, Drug Side”[tiab] OR “Side Effect, Drug”[tiab] OR “Side Effects, Drug”[tiab] OR “Adverse Drug Reaction”[tiab] OR “Adverse Drug Reactions”[tiab] OR “Drug Reaction, Adverse”[tiab] OR “Drug Reactions, Adverse”[tiab] OR “Reactions, Adverse Drug”[tiab] OR “Adverse Drug Event”[tiab] OR “Adverse Drug Events”[tiab] OR “Drug Event, Adverse”[tiab] OR “Drug Events, Adverse”[tiab] OR “Drug Toxicity”[tiab] OR “Toxicity, Drug”[tiab] OR “Drug Toxicities”[tiab] OR “Toxicities, Drug”[tiab] OR immune-related adverse event*[tiab] OR adverse event* [tiab]) AND (“Treatment Outcome”[MeSH Terms] OR “Outcome, Treatment”[tiab] OR “Patient-Relevant Outcome”[tiab] OR “Outcome, Patient-Relevant”[tiab] OR “Outcomes, Patient-Relevant”[tiab] OR “Patient Relevant Outcome”[tiab] OR “Patient-Relevant Outcomes”[tiab] OR “Clinical Effectiveness”[tiab] OR “Effectiveness, Clinical”[tiab] OR “Treatment Effectiveness”[tiab] OR “Effectiveness, Treatment”[tiab] OR “Rehabilitation Outcome”[tiab] OR “Outcome, Rehabilitation”[tiab] OR “Treatment Efficacy”[tiab] OR “Efficacy, Treatment”[tiab] OR “Clinical Efficacy”[tiab] OR “Efficacy, Clinical”[tiab] OR “survival”[tiab] OR “prognosis”[tiab] OR “prognostic”[tiab] OR “efficacy” [tiab] OR “outcome” [tiab]) AND (“Early” [tiab] OR “Earlier” [tiab] OR “early-onset”).

Supplementary Table S2. The Newcastle-Ottawa Scale (NOS) quality assessment of the enrolled studies.

Study ID	SELECTION				COMPARABILITY	OUTCOME			Total
	Representative-ness of the exposed cohort	Selection of the non-exposed cohort	Ascertain-ment of exposure	Ascertainment that outcome of interest was not present at start of study	Comparability of cohorts on the basis of design or analysis	Assessment of outcome	Was follow-up long enough for outcomes to occur	Adequacy of follow-up of cohorts	
Aso, 2020	b*	a*	a*	b	a*+b*	a*	a*	a*	8
Haratani, 2018	b*	a*	a*	b	a*	a*	b	b*	6
Hosoya,2020	b*	a*	a*	a*	a*+b*	a*	b	d	7
Ricciuti, 2019	b*	a*	a*	b	a*	a*	a*	d	6
Cortellini, 2019	b*	a*	a*	b	a*+b*	a*	b	b*	7
Ahn, 2019	b*	a*	a*	b	a*+b*	a*	b	b*	7
Verzoni, 2019	b*	a*	a*	b	a*+b*	a*	b	d	6
Ishihara, 2019	b*	a*	a*	b	a*+b*	a*	b	d	6
Masuda,2019	b*	a*	a*	b	a*	a*	a*	d	6
Teraoka,2017	b*	a*	a*	a*	a*+b*	a*	b	b*	7
Fukihara,2019	b*	a*	a*	b	a*+b*	a*	b	b*	7

* represents one point is scored

A

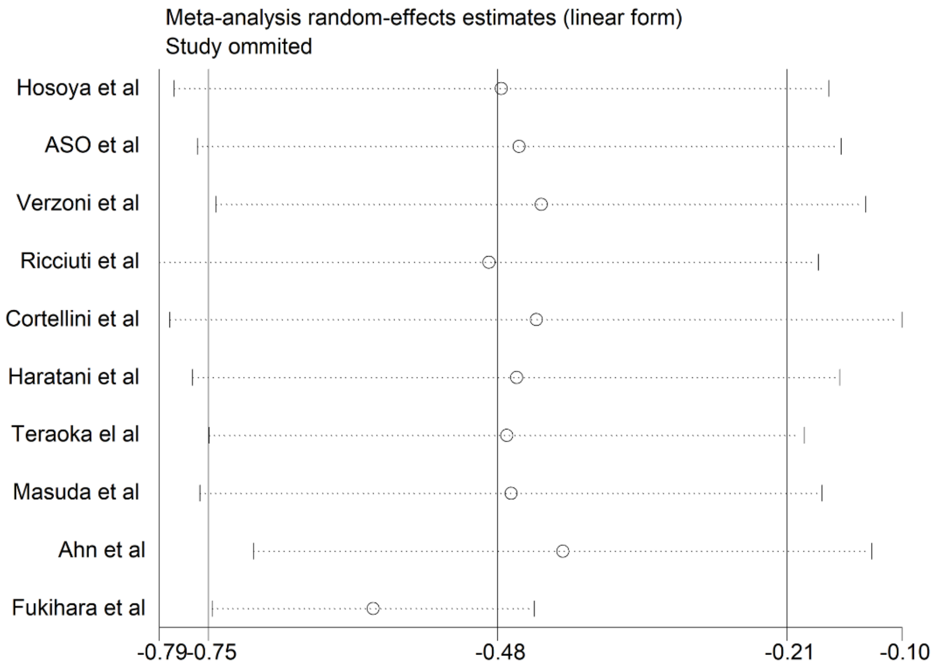

B

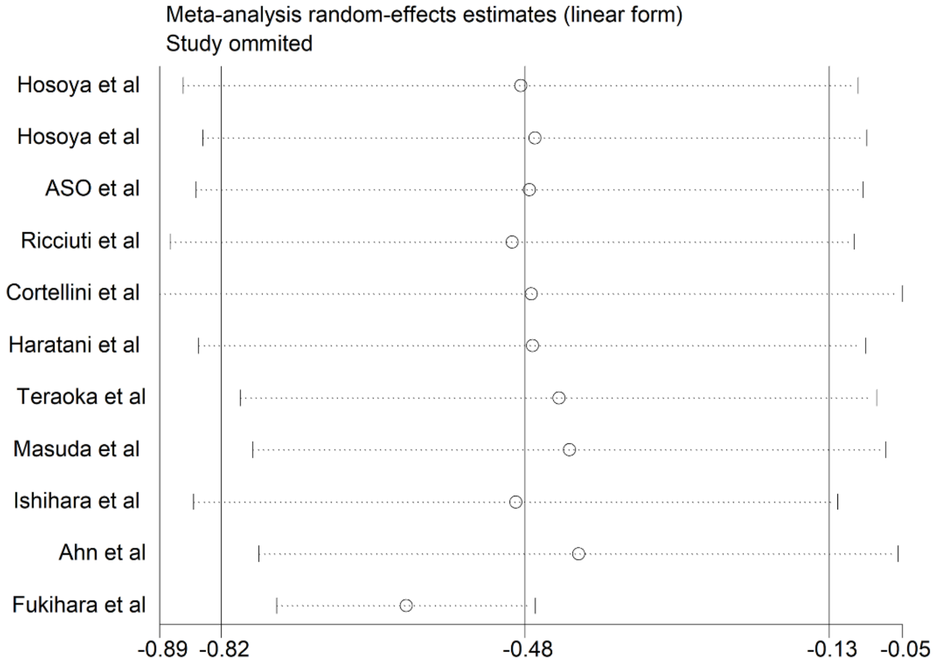

Supplementary Figure S1. The sensitivity analysis of the impact of each individual study on the pooled effect. A, Overall survival; B, progression-free survival.

A

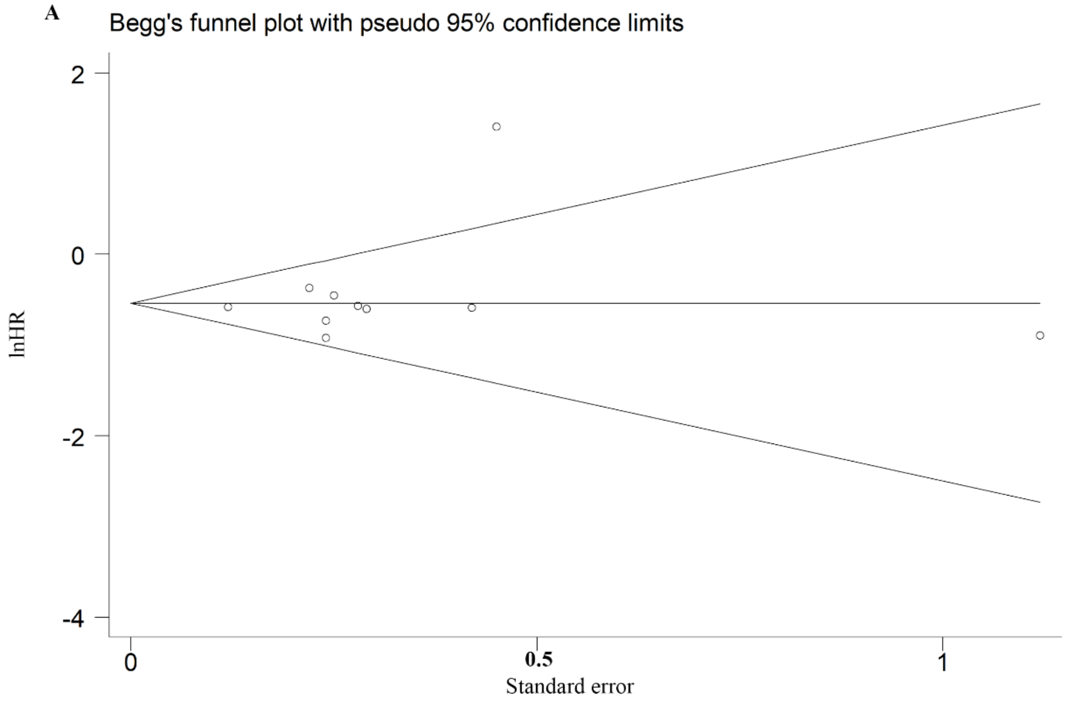

B

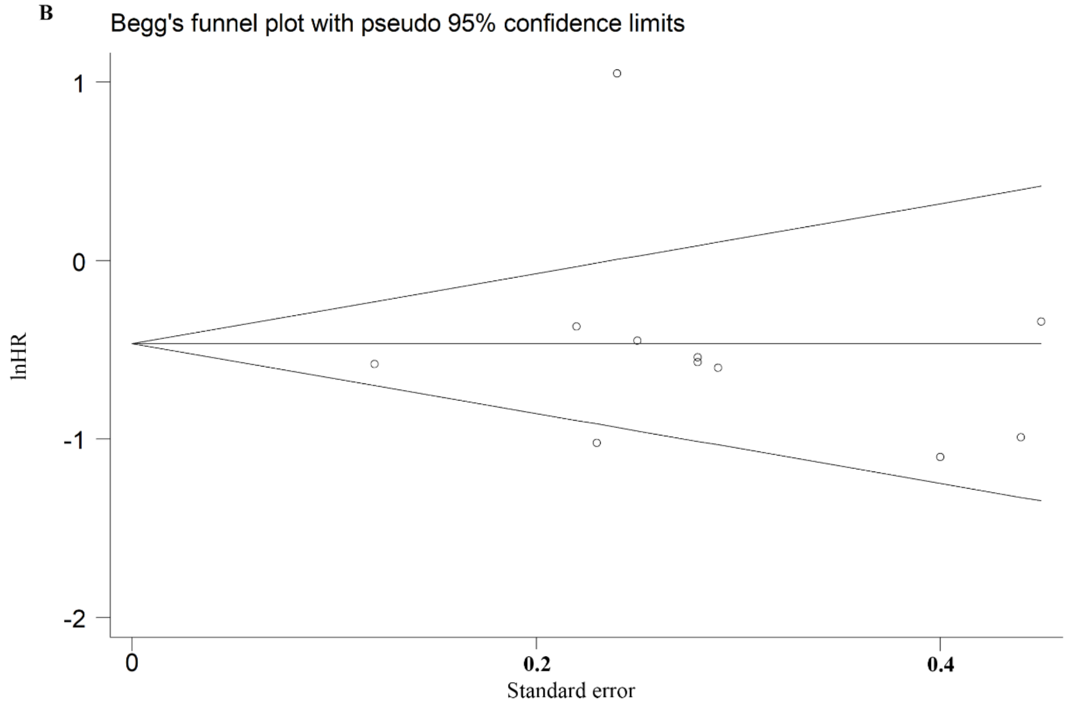

Supplementary Figure S2. Funnel plots of the overall survival (A) and progression-free survival (B)
